# Globe-LFMC 2.0, an enhanced and updated dataset for live fuel moisture content research

**DOI:** 10.1038/s41597-024-03159-6

**Published:** 2024-04-04

**Authors:** Marta Yebra, Gianluca Scortechini, Karine Adeline, Nursema Aktepe, Turkia Almoustafa, Avi Bar-Massada, María Eugenia Beget, Matthias Boer, Ross Bradstock, Tegan Brown, Francesc Xavier Castro, Rui Chen, Emilio Chuvieco, Mark Danson, Cihan Ünal Değirmenci, Ruth Delgado-Dávila, Philip Dennison, Carlos Di Bella, Oriol Domenech, Jean-Baptiste Féret, Greg Forsyth, Eva Gabriel, Zisis Gagkas, Fatma Gharbi, Elena Granda, Anne Griebel, Binbin He, Matt Jolly, Ivan Kotzur, Tineke Kraaij, Agnes Kristina, Pınar Kütküt, Jean-Marc Limousin, M. Pilar Martín, Antonio T. Monteiro, Marco Morais, Bruno Moreira, Florent Mouillot, Samukelisiwe Msweli, Rachael H. Nolan, Grazia Pellizzaro, Yi Qi, Xingwen Quan, Victor Resco de Dios, Dar Roberts, Çağatay Tavşanoğlu, Andy F. S. Taylor, Jackson Taylor, İrem Tüfekcioğlu, Andrea Ventura, Nicolas Younes Cardenas

**Affiliations:** 1 Fenner School of Environment & Society, Australian National University, Canberra, ACT Australia; 2 School of Engineering, Australian National University, Canberra, ACT, Australia; 3 ONERA / DOTA, Université de Toulouse, F-31055 Toulouse, France; 4 Department of Biology, Kastamonu University, Kastamonu, Türkiye; 5 School of Environment and Life Sciences, University of Salford, Salford, UK; 6 Faculty of Arts and Humanities, Geography Department, Tishreen University, Tishreen, Syria; 7 Department of Biology and Environment, University of Haifa at Oranim, Kiryat Tivon, 36066 Israel; 8 Instituto Nacional de Tecnología Agropecuaria, Buenos Aires, Argentina; 9 Hawkesbury Institute for the Environment, Western Sydney University, Penrith, NSW Australia; 10 University of Wollongong, Wollongong, NSW Australia; 11 US Forest Service, Rocky Mountain Research Station, Fire Sciences Laboratory, 5775 Highway 10 West, Missoula, 59803 MT USA; 12 Servei de Prevenció d’Incendis Forestals (Generalitat de Catalunya), Santa Perpètua de Mogoda, Barcelona, Spain; 13 School of Resources and Environment, University of Electronic Science and Technology of China, Sichuan, China; 14 Department of Geology, Geography and the Environment, University of Alcalá, Colegios 2, 28801 Alcalá de Henares, Spain; 15 Division of Ecology, Department of Biology, Hacettepe University, Beytepe, Ankara Türkiye; 16 Joint Research Unit CTFC - AGROTECNIO, Crta. de St. Llorenç de Morunys, km 2, E, 25280 Solsona, Spain; 17 Department of Evolutionary and Environmental Biology, University of Haifa, Haifa, Israel; 18 Department of Geography, University of Utah, Salt Lake City, Utah USA; 19 IFEVA-CONICET, Faculty of Agronomy, University of Buenos Aires, Buenos Aires, Argentina; 20 Centre Forestal de les Illes Balears (CEFOR-Menut), Forest Management Service (Government of the Balearic Islands), Palma de Mallorca, Spain; 21 INRAE, UMR TETIS, 500 rue Jean-François Breton, 34093 Montpellier, France; 22 CSIR, NRE, Stellenbosch, South Africa; 23 Environmental and Biochemical Sciences Department, The James Hutton Institute, Aberdeen, UK; 24 Faculty of Sciences of Tunis, University of Tunis El Manar, Tunis, Tunisia; 25 Departamento de Ciencias de la Vida, Universidad de Alcalá, Alcalá de Henares, Spain; 26 School of Life Sciences, University of Technology Sydney, PO Box 123 Broadway, Ultimo, NSW 2007 Australia; 27 RMRS, Missoula Fire Sciences Laboratory, USFS, Rocky Mountain Research Station, 5775 Hwy 10 W Missoula, Missoula, MT 59808 USA; 28 Nelson Mandela University, School of Natural Resource Management, George, South Africa; 29 Bushfire Technical Services, DFES WA, Perth, Australia; 30 CEFE, Univ Montpellier, CNRS, EPHE, IRD, Montpellier, France; 31 Environmental Remote Sensing and Spectroscopy Laboratory (SpecLab), IEGD, Spanish National Research Council (CSIC), Madrid, Spain; 32 Centro de Estudos Geográficos (CEG) and Laboratório Associado TERRA, Instituto de Geografia e Ordenamento do Território (IGOT), Universidade de Lisboa, Rua Edmée Marques, 1600-276 Lisboa, Portugal; 33 Istituto di Geoscienze e Georisorse, Consiglio Nazionale delle Ricerche (CNR-IGG), Via Moruzzi 2, 56124 Pisa, Italy; 34 Department of Geography, University of California, Santa Barbara, USA; 35 Department of Ecology and Global Change. Centro de Investigaciones sobre Desertificación (CIDE-CSIC/UV/GV). Carretera Moncada-Náquera km 4, 5 s/n, E-46113 Moncada, Valencia Spain; 36 IRD, CEFE/CNRS, 1919 Route de Mende, 34293 Montpellier, Cedex 5 France; 37 Natural Resource Science and Management Cluster, Nelson Mandela University, George, South Africa; 38 Istituto per la Bioeconomia, Consiglio Nazionale delle Ricerche, (CNR-IBE), Traversa La Crucca 3, 07100 Sassari, Italy; 39 University of Nebraska-Lincoln, Lincoln, Nebraska USA; 40 University of Southern California, Los Angeles, California USA; 41 Universitat de Lleida, Lleida, Spain; 42 Ecological Sciences Department. The James Hutton Institute, Aberdeen, UK

**Keywords:** Ecophysiology, Natural variation in plants, Plant physiology

## Abstract

Globe-LFMC 2.0, an updated version of Globe-LFMC, is a comprehensive dataset of over 280,000 Live Fuel Moisture Content (LFMC) measurements. These measurements were gathered through field campaigns conducted in 15 countries spanning 47 years. In contrast to its prior version, Globe-LFMC 2.0 incorporates over 120,000 additional data entries, introduces more than 800 new sampling sites, and comprises LFMC values obtained from samples collected until the calendar year 2023. Each entry within the dataset provides essential information, including date, geographical coordinates, plant species, functional type, and, where available, topographical details. Moreover, the dataset encompasses insights into the sampling and weighing procedures, as well as information about land cover type and meteorological conditions at the time and location of each sampling event. Globe-LFMC 2.0 can facilitate advanced LFMC research, supporting studies on wildfire behaviour, physiological traits, ecological dynamics, and land surface modelling, whether remote sensing-based or otherwise. This dataset represents a valuable resource for researchers exploring the diverse LFMC aspects, contributing to the broader field of environmental and ecological research.

## Background & Summary

Live Fuel Moisture Content (LFMC), a critical parameter in fire-related research, quantifies the vegetation water content. It is computed as:

$${\rm{LFMC}}\left[ \% \right]=\left({{\rm{W}}}_{{\rm{f}}}-{{\rm{W}}}_{{\rm{d}}}\right)/{{\rm{W}}}_{{\rm{d}}}\times 100$$

where W_f_ represents the weight of fresh plant material, measured post-sample collection, W_d_ indicates the weight of the same sample after thorough drying, often in an oven.

Numerous studies have demonstrated LFMC’s influence on various wildfire metrics, including flammability, rate of spread, fire occurrence and cumulative burnt area^1–5^. Growing interest surrounds the exploration of LFMC dynamics in relation to ecological, meteorological and ecophysiological parameters^6–10^, especially within the context of a changing climate^11^.

However, conducting fieldwork, collecting measurements, and recording data can be costly, time consuming, and resource-intensive. Therefore, the convenience of having access to a readily available LFMC dataset proves beneficial for advancing research. As a result, several LFMC datasets^12,13^, including the 2019 version of Globe-LFMC^14^, have emerged online.

Globe-LFMC 2.0^15^, presented herein and accessible at the *figshare* repository, represents an updated version of the 2019 release. It incorporates previously published datasets and adds more than 120,000 additional measurements hitherto unavailable to the research community.

**Fig. 1:**
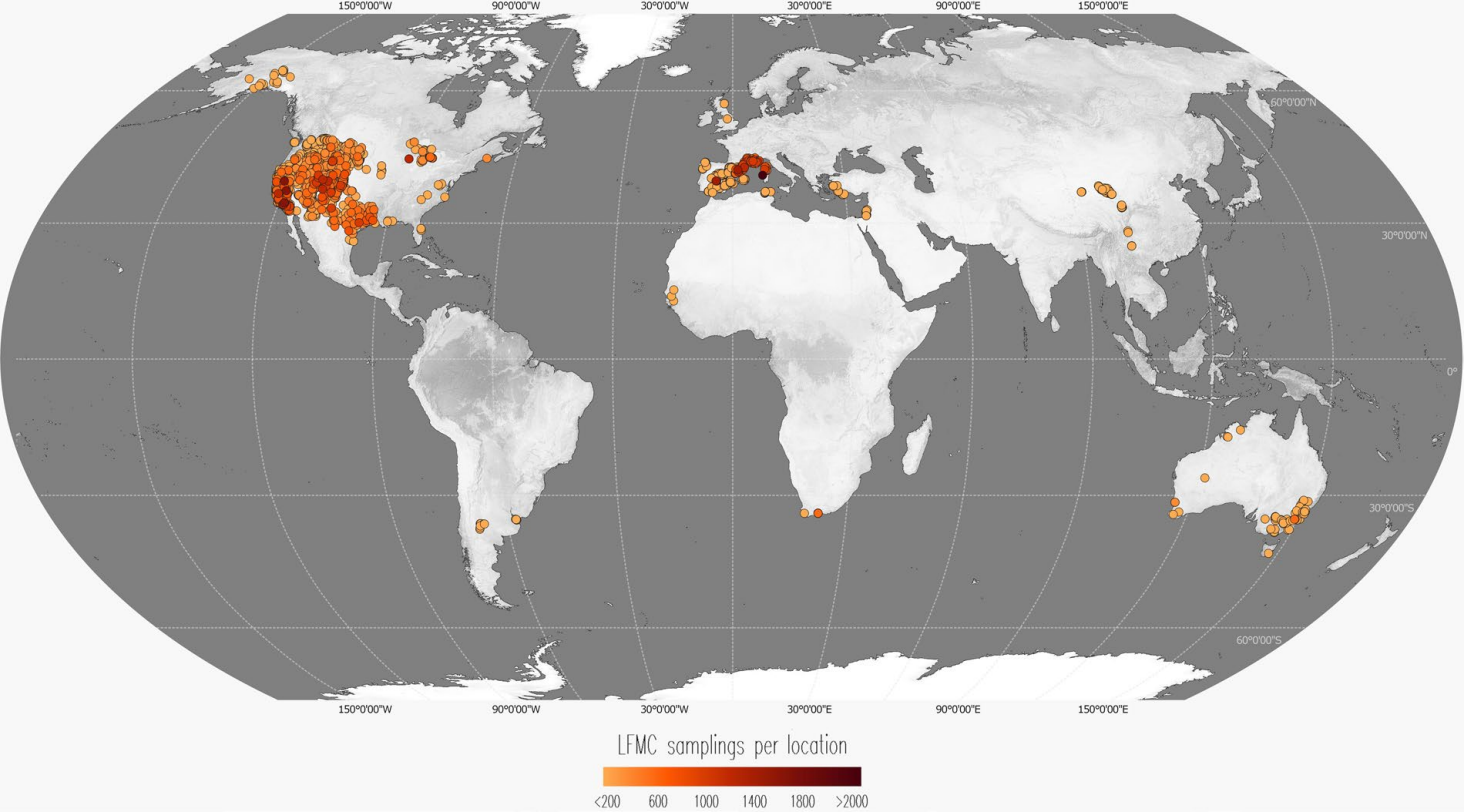
Locations of sampling sites. The sampling sites are represented as coloured points on the map, with the colour intensity indicating the abundance of LFMC values collected at each location. To enhance clarity, points have been ordered on the z-axis based on the number of LFMC samples, with sites having fewer samples placed beneath those with a higher data count. Predominantly, the sampling sites and LFMC measurements are concentrated in the USA, France, and Spain. The base map for this figure is derived from NASA’s Visible Earth ‘Explorer Base Map’^30^.

This extensive dataset comprises over 280,000 LFMC values derived from samples gathered at more than 2,000 locations across 15 countries. It includes data from more than 500 different species or combinations of species. The timeframe of the data spans from 1977 to 2023 (Tables 1, 2, Fig. 1).

**Table 1:** Metadata and Descriptive Statistics by Country (part 1 of 2). Prior to generating the descriptive statistics, outliers identified using the Isolation Forest method were excluded. The “Observations” column presents the count of LFMC values. “Sites” denotes the number of distinct sampling locations, while “Years Range” provides the span of years during which LFMC measurements were collected. In the “Most Common Species” category, we list the three species with the highest number of LFMC measurements. In cases where more than three species share the same number of observations, we organise the list alphabetically and trim to the first three names to streamline the species report. Combinations of species were treated as a single species name.

Country	Observations	Sites	Years Range	Most Common Species	Land Cover Types (IGBP)	LFMC Min	LFMC 1st Quartile	LFMC Median	LFMC 3rd Quartile	LFMC Max	LFMC Mean
Global	287551	2231	1977–2023	*Adenostoma fasciculatum; Pinus ponderosa; Artemisia tridentata ssp. wyomingensis*	Grasslands; Woody Savannas; Savannas	0.00	78.25	97.00	119.25	1278.87	105.79
Argentina	227	17	2008–2010	Unidentified grass; *Prosopis alpataco; Condalia microphylla*	Grasslands; Open Shrublands; Savannas	6.94	57.67	79.78	115.11	377.16	92.60
Australia	3227	283	2005–2022	*Eucalyptus tereticornis; Banksia sp.; Calothamnus sp*.	Woody Savannas; Grasslands; Evergreen Broadleaf Forests	5.90	70.50	97.75	124.57	627.98	102.17
China	257	226	2013–2021	*Kobresia humilis, Kobresia pygmaea, Kobresia tibetica; Pinus yunnanensis;* Unidentified grass	Grasslands; Evergreen Broadleaf Forests; Woody Savannas	52.37	134.10	166.17	201.99	323.44	170.73
England	24	6	2008–2017	*Calluna vulgaris*	Savannas; Woody Savannas	64.00	90.61	115.03	133.45	145.90	111.86
France	23788	85	1996–2022	*Cistus monspeliensis; Erica arborea; Rosmarinus officinalis*	Woody Savannas; Evergreen Needleleaf Forests; Savannas	17.14	63.86	76.80	94.56	211.53	80.10
Israel	2165	30	2018–2019	*Rhamnus alaternus; Ephedra foeminea; Pistacia palaestina*	Grasslands; Croplands; Savannas	2.49	58.56	69.64	83.93	185.97	73.38
Italy	2591	1	2005–2012	*Pistacia lentiscus; Rosmarinus officinalis; Juniperus phoenicea*	Evergreen Needleleaf Forests; Woody Savannas	34.12	75.68	97.18	118.50	236.97	100.50

**Table 2:** Metadata and Descriptive Statistics by Country (part 2 of 2).

Country	Observations	Sites	Years Range	Most Common Species	Land Cover Types (IGBP)	LFMC Min	LFMC 1st Quartile	LFMC Median	LFMC 3rd Quartile	LFMC Max	LFMC Mean
Portugal	51	20	2016–2016	Unidentified grass; *Quercus pyrenaica, Quercus robur; Betula alba*	Woody Savannas; Savannas	10.29	60.11	79.17	143.94	378.87	107.67
Scotland	250	6	2019–2021	*Calluna vulgaris*; Unidentified moss and litter	Savannas	13.07	92.18	107.63	190.98	418.46	140.51
Senegal	94	3	2010–2010	Unidentified grass	Grasslands; Croplands	14.25	76.81	105.95	174.20	327.35	129.60
South Africa	922	57	2016–2018	*Erica spp., Metalasia spp., Brunia spp., Berzelia spp., Agathosma spp., Cliffortia spp.; Leucadendron spp., Penaea sp., Protea spp.; Cyperaceae spp., Restionaceae spp*.	Evergreen Broadleaf Forests; Closed Shrublands; Savannas	45.41	99.66	115.91	148.20	637.97	131.32
Spain	18338	183	1996–2021	*Quercus coccifera; Salvia rosmarinus; Pinus halepensis*	Grasslands; Woody Savannas; Savannas	0.18	71.35	94.88	117.88	1278.87	107.65
Tunisia	430	10	2010–2022	*Erica arborea; Cistus monspeliensis; Quercus suber*	Croplands; Evergreen Needleleaf Forests; Evergreen Broadleaf Forests	26.18	75.85	94.27	117.86	220.01	98.51
Türkiye	661	45	2017–2022	*Quercus coccifera; Cistus creticus; Pinus brutia*	Savannas; Grasslands; Woody Savannas	15.03	94.20	125.42	182.95	861.54	148.24
USA	234526	1259	1977–2023	*Adenostoma fasciculatum; Pinus ponderosa; Artemisia tridentata ssp. wyomingensis*	Grasslands; Woody Savannas; Savannas	0.00	82.00	99.00	122.00	887.00	108.35

**Fig. 2:**
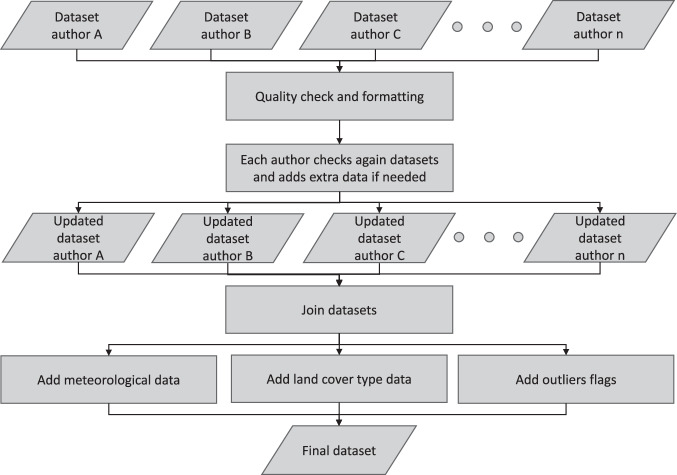
Workflow followed to compile Globe-LFMC 2.0^15^.

The compilation process included formatting source data, performing rigorous and recursive quality checks, merging data from co-authors, and introducing supplementary information. Notably, each data point now includes land cover type and meteorological variables, aligned with the sampling date and location. An outlier detection analysis was executed, and its findings are presented (Fig. 2).

Distinguishing Globe-LFMC 2.0^15^ from its predecessor, it presents two significant enhancements. First, it incorporates a large number of LFMC measurements from individual samples, broadening its coverage across various geographic and climatic conditions. Second, it includes additional descriptor variables per sample (Tables 3, 4) and rectifies inaccuracies and typos that may have been present in the previous version. These improvements not only increase the comprehensiveness of the dataset but also enhance its adaptability for end-users, allowing them to process the data and aggregate the samples as they see fit.

Globe-LFMC 2.0^15^ applications are manifold. Researchers can employ it to develop and validate models for LFMC estimation from remote sensing data^16,17^, or for other types of land surface modelling, such as those derived from climate variables^3^. It is equally valuable in investigating the relationship between LFMC and wildfire occurrence and behaviour, as well as its associations with other plant water status metrics, meteorological parameters and ecological drivers.

In conclusion, as we plan to keep the dataset updated and publish future versions, we invite researchers and other interested parties to contact us if they wish to contribute.

## Methods

### Compilation of Live Fuel Moisture Content measurements

Globe-LFMC 2.0^15^ is the result of collaborative efforts involving international researchers and agencies, incorporating data from multiple sources, including publicly available datasets^12–14,18,19^.

The authors meticulously adapted their datasets to conform to the template spreadsheets, aligning with the structured format of Globe-LFMC 2.0^15^ (a comprehensive breakdown of the dataset fields is available in the Data Records section). These refined spreadsheets were subsequently integrated into a unified dataset, following a rigorous visual quality check. This check was essential to verify data integrity, and rectify any typographical errors, formatting inconsistencies and obsolete information to ensure the dataset’s reliability and accuracy.

LFMC values in this dataset were derived from destructive measurements of plant materials obtained during field sampling. While sampling and weighing protocols varied among contributors, the common procedure involved weighing fresh plant material, typically leaves, either in the field or a laboratory after secure transportation in a sealed bag or container. Subsequently, the samples were oven-dried for several hours at a minimum of 60 °C and re-weighed. Sampling details, including location, date, and sometimes the time of sampling, as well as specific sampling protocols, were meticulously recorded.

Unlike the previous version of Globe-LFMC^14^, efforts were made to avoid data aggregation and preserve individual sample measurements wherever possible. This means that values corresponding to the same combination of species, sampling location, and date were not averaged together. In cases where data from the 2019 version of the dataset were included, averaging was replaced with the original individual measurements, when available.

Entries that remained as mean LFMC values for multiple measurements were flagged in a dedicated dataset column.

A comprehensive review of the 2019 dataset was undertaken to rectify typos and inaccuracies, encompassing species names, protocol details, and, in a limited number of instances, sampling dates (a list of the dates changed is available at the *figshare* repository^15^).

The US National Fuel Moisture Database (NFMD)^19^ was redownloaded from the original source, leading to differences from the previous Globe-LFMC^14^ version. Some data entries were added, others were removed. Dead Fuel Moisture measurements were excluded, while all LFMC values were retained, irrespective of whether they were later identified as outliers during the quality check. The decision not to delete these values was due to impracticality in contacting the original data providers for further investigation.

After compiling all data sources, extensive efforts were made to harmonize the diverse datasets, ensuring uniformity and consistency across Globe-LFMC 2.0^15^. In cases where the same site name was associated with different coordinates, we introduced unique identifiers at the end of the name to distinguish them. Conversely, when identical coordinates were linked to multiple sites, their names were merged. This meticulous process culminated in a dataset where each site name corresponded exclusively to one set of coordinates, and vice versa, fostering data integrity and precision.

### Land cover data

Land cover type information was also added to the final dataset following the IGBP classification from LP DAAC MCD12Q1.061 (MODIS/Terra + Aqua Land Cover Type Yearly L3 Global 500 m SIN Grid)^20^.

The process started by downloading the complete set of MCD12Q1.061 sinusoidal tiles products spanning the years 2001 to 2022. Subsequently, these tiles were mosaicked into yearly raster images at a spatial resolution of 500 m within the WGS84 reference system.

For each LFMC value, the mosaic corresponding to the respective calendar year was employed to retrieve the land cover ID by selecting the pixel value at the precise sampling location. Additionally, the descriptive land cover name (e.g., “Grasslands”) was incorporated into the dataset.

Given that the available land cover time series extended from 2001 to 2022, the land cover type of 2001 was attributed to all samples collected before 2001, as it most closely represented the respective sampling date. Similarly, for samples collected after 2022, the land cover type of the year 2022 was assigned. This method ensured consistent land cover information across all samples.

### Meteorological data

Meteorological data was sourced from AgERA5 (Agrometeorological indicators from 1979 to present derived from reanalysis) AgERA5 is a high level product built upon ERA5 data, which were aggregated to obtain daily values and downscaled to 0.1° × 0.1° spatial resolution^21^.

The initial step involved downloading NetCDF files containing specific meteorological variables: total daily precipitation, relative humidity at 2 m above surface at four distinct times (6am, 9am, 12 pm and 3 pm), maximum daily air temperature at 2 m above surface, mean daily air temperature, mean daily vapour pressure, mean daily wind speed at 10 m above surface and mean daily dewpoint temperature at 2 m above surface.

Subsequently, the values for each meteorological variable were extracted from the downloaded files at the date and location of each entry in the dataset.

Additionally, cumulative precipitation data for the preceding 3 days, 1 week, 4 weeks, and 12 weeks before the sampling date was included in the final dataset.

### Detection of possible outliers

The process of identifying potential outliers within LFMC values consisted of a two-step strategy, combining both manual inspection and the application of two distinct statistical models.

We define outliers as values that deviate notably from the norm, being either anomalously high or low. Such deviations may arise from measurement inaccuracies due to instrument or human errors. Additional context regarding the interpretation of outlier detection is available in the “Technical Validation” section.

Step 1: Manual Inspection and Data Provider-Specific Methods

In the initial phase, when possible, data providers meticulously examined each dataset comprising Globe-LFMC 2.0^15^. Since these datasets varied significantly in structure, the authors customized outlier detection methods for each. The outcomes of this initial assessment were documented in the “Extra information/Quality Flag” column of the dataset. The methods used in this step were tailored to the specific dataset’s characteristics, involving visual inspections, percentile-based, or standard deviation-based approaches to identify outliers.

Step 2: Statistical Model-Based Outlier Detection

**Fig. 3:**
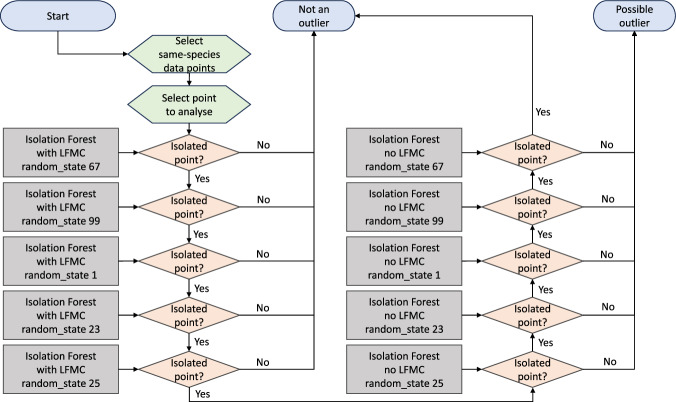
Decision diagram explaining the outlier detection method based on the Isolation Forest algorithm.

The second approach leveraged the Isolation Forest algorithm^22^, a tool that utilises binary trees to identify data points as outliers via random splits in the dataset. Fewer splits required to isolate a data point indicate a higher likelihood that it is an outlier. The implementation of this method was conducted through Python’s Scikit-Learn 1.3.0 library^23^ as illustrated in Fig. 3. Isolation Forest analysis was executed on separate data subsets categorised by species. The variables integrated into the models were time, latitude, longitude, and LFMC to account for both variations among local populations of the same species and fluctuations in time series data.

Due to the unsupervised nature of this model, hyperparameters were predefined with a theoretical approach as the true anomalies were unknown. Key hyperparameters included the number of trees (“n_estimators”) set at 10,000, which was considered sufficient for building a precise model without excessive computational demands. Additionally, “max_samples” was set at 75% of each subset total data points to facilitate the detection of outlier clusters. The inclusion of bootstrap, “max_features” set at 4, and a contamination ratio of 0.05 was determined based on a conservative assessment of the data following preliminary visual examination.

A potential limitation of this approach is its propensity to identify data points as anomalies when they are isolated in time or space, even if their LFMC values are within the expected range. To minimize this risk, a complementary model was simultaneously applied to the same subsets. This model specifically focused on time, latitude, and longitude, with a “max_features” setting adjusted to 3. It aimed to detect data points isolated independently of their LFMC values. The anomalies identified by this secondary model were then subtracted from those found by the LFMC-inclusive model, producing a refined list of anomalous LFMC values.

Given the stochastic nature of the Isolation Forest algorithm, five model versions were created (both including and excluding LFMC as a variable), each employing different random states. A data point was designated as a possible outlier only if all five LFMC-inclusive models identified it as isolated, and at least one of the five models without LFMC did not isolate it, as depicted in Fig. 3. Data points isolated by all models with and without LFMC were not classified as outliers, as their isolation could be attributed to time or spatial factors unrelated to their LFMC values.

The results of all ten models, along with their respective scores, are provided in the *figshare* repository^15^.

Moreover, the repository contains results from an alternative outlier detection method: Cook’s Distance^24^, which gauges the influence of a data point on a regression line. This analysis was conducted using the Python library statsmodels 0.14.0^25^. It involved grouping data points by species and sampling location, calculating ordinary least squares regression, and comparing Cook’s Distance scores to the “4/n” threshold (where “n” stands for the number of observations within a group of samples), commonly used to identify influential data points^26,27^. An additional criterion was considered, flagging data points with Cook’s Distance values more than three times the mean Cook’s Distance of data points in the same group.

In cases where Cook’s Distance resulted in NaN (not a number) or infinite values, “NA” (not available) was assigned to all data points within the same group.

## Data Records

The Globe-LFMC 2.0 dataset^15^ is available in an MS Excel file containing three sheets: “Contact” (Table 5), “LFMC Data” (Tables 3, 4) and “Protocol” (Table 6). The primary dataset is located within the “LFMC Data” sheet, which contains the core LFMC values along with associated information. The “Contact” sheet offers supplementary details regarding the contact person responsible for each sub dataset, facilitating direct communication and inquiries related to the data. In the “Protocol” sheet, a comprehensive description of the sampling and weighing procedures employed to obtain the LFMC measurements is presented, providing essential context for data interpretation.

**Table 3:** Attributes Describing the Contents of the “LFMC Data” Tab (part 1 of 2).

Field	Description
Sorting ID	Unique ID, useful for referring or point to specific rows of the dataset
Contact	Last name of the contact person for a subset. Further details in the “Contact” sheet
Site name	Name of the sampling location
Country	Country where the sample was collected
State/Region	State or region where the sample was collected
Latitude (WGS84, EPSG:4326)	Latitude of the sampling location (WGS84 decimal degrees)
Longitude (WGS84, EPSG:4326)	Longitude of the sampling location (WGS84 decimal degrees)
Sampling date (YYYYMMDD)	Date when the sample was collected (in the format YYYYMMDD)
Sampling time (24 h format)	Time when the sample was collected (in the format hh:mm)
Protocol	ID of sampling and weighing protocol. Detailed procedures reported in the “Protocol” sheet
LFMC value (%)	Live Fuel Moisture Content value, calculated as (Wf - Wd)/Wd × 100, where Wf is the weight of the fresh sample and Wd is the weight of the same sample after being dried
Species collected	Scientific name of the species sampled
Species functional type	High level functional type of vegetation sampled (e.g., tree, shrub, grass)
Individual sample or mean value	Whether the LFMC value was obtained by measuring an individual sample or by computing the mean of LFMC values from multiple samples (simple average, unless specified)
Old or new leaves	Whether the sample was composed by old growth or new growth plant material
Elevation (m.a.s.l)	Elevation in metres of the sampling location
Slope (%)	Percentage slope of the sampling location
Extra information/Quality Flag	Optional extra information beneficial in interpreting and using the data. The information was added by the providers of the data and it is unique to each subset (i.e., it is not consistent across the whole dataset)
Isolated data point	True if a data point was identified as isolated by the combination of Isolation Forest models (details in the method section). This column can be used to further investigate values that could be outliers
Reference	Citation of relevant studies that employed the data or of source datasets and their licence
Name of picture file	Name of the photograph showing the sampling site

**Table 4:** Attributes Describing the Contents of the “LFMC Data” Tab (part 2 of 2).

Field	Description
IGBP Land Cover	Land cover type according to the International Geosphere-Biosphere Programme (IGBP) classification (from LP DAAC MCD12Q1.061, 10.5067/MODIS/MCD12Q1.061)
IGBP Land Cover ID	Integer ID of land cover type according to the International Geosphere-Biosphere Programme (IGBP) classification (from LP DAAC MCD12Q1.061, 10.5067/MODIS/MCD12Q1.061)
Year of Land Cover	Year considered for land cover classification (available range of years: 2001–2022 inclusive. The land cover of the year 2001 was also assigned to all years prior to 2001, while the land cover of the year 2022 was also assigned to all years after 2022)
Precipitation 24 h sum (mm/day)	Total daily (24 hours, local time) precipitation (from AgERA5, 10.24381/cds.6c68c9bb, 0.1° × 0.1° spatial resolution)
Precipitation sum 3 days before (mm/day)	Aggregate precipitation of the 3 days prior to sample collection (from AgERA5, 10.24381/cds.6c68c9bb, 0.1° × 0.1° spatial resolution)
Precipitation sum 1 week before (mm/day)	Aggregate precipitation of the 7 days prior to sample collection (from AgERA5, 10.24381/cds.6c68c9bb, 0.1° × 0.1° spatial resolution)
Precipitation sum 4 weeks before (mm/day)	Aggregate precipitation of the 28 days prior to sample collection (from AgERA5, 10.24381/cds.6c68c9bb, 0.1° × 0.1° spatial resolution)
Precipitation sum 12 weeks before (mm/day)	Aggregate precipitation of the 84 days prior to sample collection (from AgERA5, 10.24381/cds.6c68c9bb, 0.1° × 0.1° spatial resolution)
2 m Relative Humidity at 06 h (%)	Relative humidity at 6:00am local time and at 2 m above the surface (from AgERA5, 10.24381/cds.6c68c9bb, 0.1° × 0.1° spatial resolution)
2 m Relative Humidity at 09 h (%)	Relative humidity at 9:00am local time and at 2 m above the surface (from AgERA5, 10.24381/cds.6c68c9bb, 0.1° × 0.1° spatial resolution)
2 m Relative Humidity at 12 h (%)	Relative humidity at 12:00 pm local time and at 2 m above the surface (from AgERA5, 10.24381/cds.6c68c9bb, 0.1° × 0.1° spatial resolution)
2 m Relative Humidity at 15 h (%)	Relative humidity at 3:00 pm local time and at 2 m above the surface (from AgERA5, 10.24381/cds.6c68c9bb, 0.1° × 0.1° spatial resolution)
2 m Air Temperature 24 h max (K)	Maximum daily (24 hours, local time) air temperature at 2 m above the surface (from AgERA5, 10.24381/cds.6c68c9bb, 0.1° × 0.1° spatial resolution)
2 m Air Temperature 24 h mean (K)	Mean daily (24 hours, local time) air temperature at 2 m above the surface (from AgERA5, 10.24381/cds.6c68c9bb, 0.1° × 0.1° spatial resolution)
Vapour Pressure 24 h mean (hPa)	Mean daily (24 hours, local time) vapour pressure (from AgERA5, 10.24381/cds.6c68c9bb, 0.1° × 0.1° spatial resolution)
10 m Wind Speed 24 h mean (m/s)	Mean daily (24 hours, local time) wind speed at 10 m above the surface (from AgERA5, 10.24381/cds.6c68c9bb, 0.1° × 0.1° spatial resolution)
2 m Dewpoint Temperature 24 h mean (K)	Mean daily (24 hours, local time) dewpoint temperature at 2 m above the surface (from AgERA5, 10.24381/cds.6c68c9bb, 0.1° × 0.1° spatial resolution)

**Table 5:** Attributes Describing the Contents of the “Contact” Tab Table.

Field	Description
First name	First name of the contact person
Last name	Last name of the contact person
Email	Contact e-mail address
Phone (include all codes)	Contact phone number
Collaborations	People who contributed to the data collection
Institution	Institution or company where the contact person works
Address	Address of the institution
Country	Country of the institution
Web page	URL to the institutional profile webpage or personal professional webpage

**Table 6:** Attributes Describing the Contents of the “Protocol” Tab.

Field	Description
Protocol code	ID corresponding to the protocol reported in the “LFMC data” tab
Time range for sampling	Range of time designated for field work (i.e., sample collection)
New and old leaves combined	Whether the Fuel Moisture Content value is a combination of old growth and new growth leaves
Fresh samples weighing location	Whether the sample was weighed at the field or at the laboratory
Weighing device accuracy (g)	Accuracy (g) of the weighing device used
Material for transportation	Material or equipment used to carry the sample to the laboratory
Drying time (h)	Duration (hours) of the drying procedure of the sample
Drying temperature (°C)	Temperature (Celsius degrees) which the sample was dried at
OBSERVATIONS	Further comments and information regarding the protocol used

Accompanying the dataset, additional files are provided for reference and extra data. In these files it is possible to retrieve all the outcomes generated from the outlier detection procedures, offering transparency and insight into data quality control, as well as the references to the original sources and datasets incorporated into Globe-LFMC 2.0^15^. The files are equipped with column descriptions where needed, enhancing the accessibility and usability of the dataset.

## Technical Validation

A rigorous quality check of the LFMC data was conducted individually by each contributing author, as outlined in the Methods section. Furthermore, to ensure data integrity, two outlier detection methods, the Isolation Forest and the Cook’s Distance, were applied across the entire dataset (see Usage Notes for details).

**Fig. 4:**
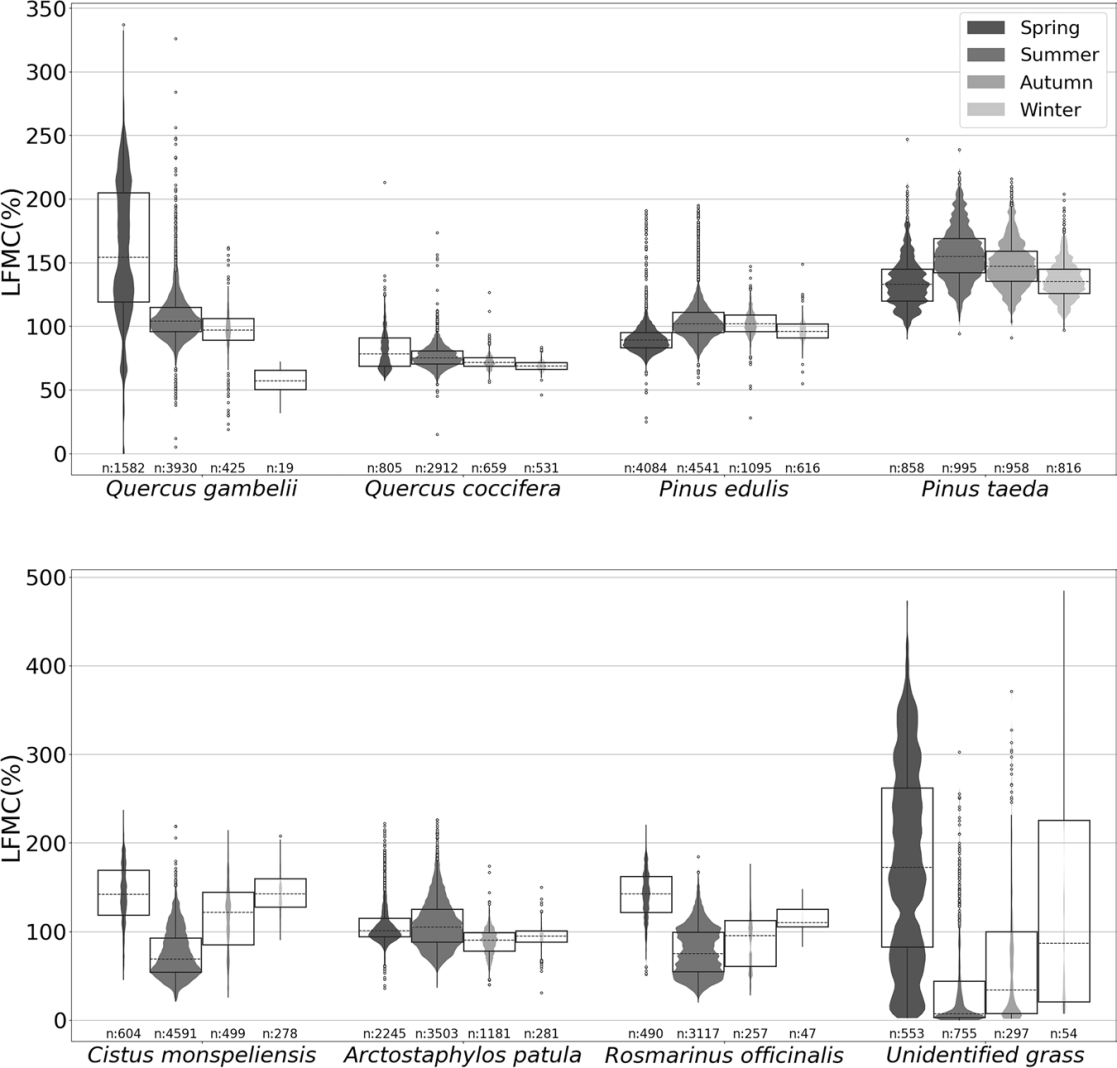
Box plots and violin plots illustrating the seasonal variability and statistical distribution of LFMC for eight common species found in Globe-LFMC 2.0^15^. The species include *Quercus gambelii*, a deciduous oak (sampled in USA); *Quercus coccifera*, an evergreen oak (sampled in France, Spain, Türkiye); *Pinus edulis*, a medium sized pine (sampled in USA); *Pinus taeda*, a tall pine (sampled from the USA); *Cistus monspeliensis*, an evergreen shrub with narrow leaves (sampled in France, Italy, Spain, Tunisia); *Arctostaphylos patula*, an evergreen shrub with round leaves (sampled in USA); *Rosmarinus officinalis*, an evergreen shrub with narrow leaves (sampled in France, Italy, Spain, Tunisia); and unidentified grass encompassing various unidentified grass species collected in grasslands (sampled in Argentina, Australia, China, Portugal, Senegal, Spain). The seasons were defined based on time ranges between astronomical equinoxes and solstices. (Figure created using seaborn^31^).

Upon removal of data points flagged as potential anomalies by the Isolation Forest method, the LFMC values generally fell within expected ranges, as demonstrated in Fig. 4 and detailed in Table 7, which provides example LFMC distributions and descriptive statistics for some of the most common species in the dataset.

**Table 7:** Metadata and descriptive statistics of the 20 most sampled species in Globe-LFMC 2.0^15^ excluding outliers identified by the Isolation Forest method.

Species	Observations	Sites	Countries	Years Range	LFMC Min	LFMC 1st Quartile	LFMC Median	LFMC 3rd Quartile	LFMC Max	LFMC Mean
*Adenostoma fasciculatum*	32434	146	USA	1977–2023	16.00	61.00	71.00	87.00	299.00	78.01
*Pinus ponderosa*	17839	280	USA	1995–2022	6.00	91.00	101.00	112.00	315.00	103.99
*Artemisia tridentata ssp. wyomingensis*	14538	118	USA	1984–2022	36.00	87.00	113.00	160.00	358.00	125.79
*Pinus edulis*	10336	118	USA	1996–2023	25.00	88.00	96.00	105.00	195.00	98.50
*Pseudotsuga menziesii*	10266	178	USA	1995–2022	8.00	93.00	105.00	120.00	387.00	108.52
*Juniperus osteosperma*	9817	91	USA	1996–2022	6.00	74.00	81.00	89.00	139.00	82.50
*Artemisia tridentata*	9733	62	USA	1984–2022	28.00	89.00	116.00	161.00	337.00	126.98
*Artemisia tridentata ssp. vaseyana*	9036	87	USA	1989–2023	13.00	93.00	123.00	166.00	284.00	130.89
*Arctostaphylos patula*	7210	103	USA	1982–2022	31.00	89.00	100.00	116.00	226.00	104.55
*Juniperus scopulorum*	7008	88	USA	1999–2022	20.00	78.00	86.00	96.00	200.00	87.90
*Pinus contorta*	6793	105	USA	1995–2022	60.00	100.00	109.00	120.00	248.00	111.80
*Cistus monspeliensis*	5972	34	France; Spain; Italy	1996–2022	21.25	57.34	79.12	112.95	236.97	88.15
*Quercus gambelii*	5956	87	USA	1995–2022	0.00	97.00	108.00	128.25	337.00	120.04
*Arctostaphylos manzanita*	5174	38	USA	1983–2023	35.00	86.00	98.00	113.00	352.00	106.61
*Quercus coccifera*	4907	69	Spain; France; Türkiye	1996–2022	15.03	69.10	73.66	80.19	213.13	75.73
*Pinus banksiana*	3933	31	USA	1995–2022	21.00	102.00	111.00	122.00	218.00	113.75
*Rosmarinus officinalis*	3911	48	France; Spain; Italy	1996–2022	20.31	58.00	84.18	108.26	220.22	87.07
*Pinus taeda*	3627	16	USA	2001–2023	90.00	130.00	143.00	157.00	247.00	144.90
*Juniperus coahuilensis*	3446	25	USA	2001–2023	46.00	78.00	89.00	100.00	180.00	89.45
*Pinus resinosa*	3422	29	USA	2001–2022	67.00	93.00	103.00	116.00	202.00	105.95

Notably low LFMC values may be attributed to samples that contain a combination of live and dead plant material or, in some cases, exclusively dead material from living vegetation. Similarly, very high LFMC values not identified as potential outliers could originate from juvenile leaves, fleshy plant species, or samples influenced by waterlogged soil conditions. Whenever available, this contextual information was included in the dataset.

It is important to acknowledge that certain data points may not have been identified as anomalies by the method depicted in Fig. 3, potentially due to isolation in time or space, irrespective of their LFMC value.

Moreover, although efforts were made to detect outliers, it is possible that a small number of very high values remain unidentified due to the stochastic nature of the method applied (Isolation Forest).

The correctness of the land cover and meteorological values added to the dataset was verified visually by comparing the output of the Python scripts with the source raster images in a Geographic Information System (GIS) software. This validation process was conducted on a small randomly selected subset consisting of 15 data points (one per country).

## Usage Notes

The “LFMC data” sheet contains various attributes that can be utilized for data filtering and categorization as per research requirements. Additionally, it offers valuable meteorological and land cover data that can support the study of LFMC dynamics. Tables 3, 4 provide detailed explanations for each column, but further guidance on how to effectively use some of the more intricate attributes is provided below.

The “Species functional type” column provides a generic classification of the sampled species. It is particularly valuable for understanding the vertical structure of the collected species within the plot, especially when different species are sampled from the same location. The functional types were assigned by data providers based on their expertise. Hence, intermediate-size plants were occasionally categorised using different terms depending upon each author’s judgement (e.g., “small tree” and “large shrub” could refer to plants having analogous size).

Functional type information is especially useful for optical remote sensing studies, particularly in closed forests, where the canopy may obstruct visibility of lower vegetation layers. In such cases, it is advisable to select only measurements from trees.

For remote sensing applications, it is recommended to average the LFMC measurements taken on the same date and located within the same pixel of the product employed in the study. The choice of which functional type to include in the average can be guided by the land cover type of that pixel. For example, in open canopy forests, both trees and shrubs (or grass) could be included.

However, caution is advised when utilising land cover information, given the 500 m spatial resolution and inherent uncertainties in the satellite-based product, which may compromise the accuracy of land cover classification.

The “protocol” column and accompanying protocol sheet can be used to filter the data based on specific research requirements. For instance, selecting only LFMC values retrieved following a specific sampling and weighing criteria or excluding samples that might have included flowers or buds.

Land cover type and meteorological data are provided to aid preliminary studies and hypothesis testing regarding LFMC dynamics or investigation of reasons behind anomalous LFMC values, or retrieval of information about the plant type sampled.

The “Extra information/Quality Flag” column contains additional miscellaneous information provided by data providers to enhance the understanding of the data. It may include markers for suspected anomalies, explanations for unusual LFMC values, or information about the plant type sampled.

“Isolated data point” reports the output of the Isolation Forest models (Fig. 3). Users can employ this column to filter the dataset by removing “isolated” data points, which could be potential outliers (by only selecting the “FALSE” values; i.e., not isolated).

Instead of removing potential outliers from the dataset, adding flags enables each user to employ the data in the way that best suits their research needs.

It is important to note that if a data point is identified as isolated (value “TRUE”), it may not necessarily be a true outlier, as the algorithm compares it only to other data points in the same subset without prior knowledge of LFMC variability of a given species.

Moreover, it is possible that anomalous LFMC values were not flagged as outliers because those data points were selected as isolated in time or space by all the models without LFMC (see Methods for details), and they were subtracted from the potential outliers.

Lastly, due to the random nature of this method, both false positives and false negatives are possible.

Further outlier detection criteria are provided in the *figshare* repository^15^, including columns reporting the results from Cook’s Distance method. The columns “Above 4/n Cook Distance” and “Above 3xMean Cook Distance” are two ready-to-use quality flags that can help identify influential data points. Cook’s Distance methods tended to detect a much higher number of outliers; hence they appear to be more conservative than the Isolation Forest. However, it can also sometimes fail to identify possible outliers with suspiciously high (or low) LFMC values, if there are other values in the same subset that are even higher (or lower).

Moreover, additional output data from both outlier detection methods are also shared, providing dataset users with the flexibility to create customized filters to suit their specific requirements. For instance, users can employ algorithms’ scores to establish custom thresholds. Alternatively, in the context of the Isolation Forest method, they can flag a data entry as a potential outlier even if does not meet the consensus of five models.

Furthermore, it is possible to employ a combination of different methods. For example, the Cook’s Distance metrics can be used to cross-verify LFMC values of data points that were not classified as anomalies by the Isolation Forest method only because they were detected as isolated in time and space.

Finally, it is strongly recommended to use the most recent version of the dataset, as it incorporates corrections for occasional inaccuracies and typos. Continued use of the 2019 version is discouraged.

## Data Availability

The code for the detection of potential outliers and the extraction of land cover and meteorological data was developed using Python 3.9.7. The corresponding Jupyter Notebooks are available at the *figshare* repository^15^. The outlier detection code uses the Globe-LFMC-2.0^15^ file as input. When executing the land cover and meteorological data extraction code, it is essential to have downloaded the required input data first.
